# Letter to the Editor: Clonal hematopoiesis of indeterminate potential and the risk of breast cancer: a UK Biobank cohort study

**DOI:** 10.1097/JS9.0000000000003200

**Published:** 2025-08-07

**Authors:** Ailun Yang, Jiajun Pan

**Affiliations:** Department of Hematology and Oncology, Dongzhimen Hospital, Beijing University of Chinese Medicine, Beijing, China

*Dear Editor,*

Breast cancer is currently the most commonly diagnosed malignancy among women. As of 2020, its incidence has even surpassed that of lung cancer, making it the most frequently diagnosed cancer globally[1]. We recently read with great interest the article by Feng *et al*, titled “Clonal hematopoiesis of indeterminate potential and the risk of breast cancer: a UK Biobank cohort study”, published in the *International Journal of Surgery*. The study provides valuable insights into the significant association between clonal hematopoiesis of indeterminate potential (CHIP) and increased breast cancer risk, highlighting the potential for genetic screening and early detection. However, several critical aspects merit further exploration. This study is compliant with the TITAN Guidelines 2025 – governing declaration and use of AI[2].

First, an important area that merits further investigation is the role of DNMT3A mutations in the tumor microenvironment (TME) of breast cancer. Chronic inflammation is a known driver of tumorigenesis and can facilitate an immunosuppressive TME through the recruitment of various immune suppressor cells, thereby promoting tumor initiation and progression[3]. DNMT3A is involved in regulating inflammatory responses; however, the specific impact of DNMT3A mutations on the composition of myeloid cells within the TME and their interactions with other immune components remains insufficiently characterized. Breast cancer is a heterogeneous solid tumor composed of diverse cell populations that interact within a complex network of cytokines[4]. Whether DNMT3A mutations directly induce breast cancer, trigger one or more inflammatory pathways, or alter the nutritional and inflammatory milieu that indirectly influences other immune elements, thus increasing cancer risk, is still unclear.

Moreover, the study does not sufficiently address the cumulative or synergistic effects of multiple CHIP mutations at the genetic level. While the analysis of individual mutations such as DNMT3A and ATM offers important insights into their correlation with breast cancer risks, the internal physiological environment is highly complex. The potential for combinatorial or additive effects of multiple CHIP-related mutations remains unexplored. Previous studies have indicated that interactions among multiple CHIP-associated mutations may exacerbate immune dysfunction and elevate malignancy risk[5]. A more comprehensive investigation is necessary to assess the broader impact of these mutations on cancer progression and immune system impairment.

Secondly, in their discussion of CHIP-related mutations, the authors referenced mutations in TET2, JAK2, and ASXL1 and further underscored their associations with diseases such as acute kidney injury and lung cancer. However, the rationale for focusing on lung cancer-related mutations, particularly in the context of breast cancer, is not entirely clear. Although mutations in genes such as ASXL1 have been well studied in lung cancer, it is essential to explore whether there are shared or convergent pathogenic mechanisms between lung and breast cancers

Lastly, translating CHIP into a viable early detection strategy for breast cancer will require precise screening criteria. Current genetic screening guidelines for breast cancer focus predominantly on hereditary germline mutations and lack CHIP-specific thresholds. Furthermore, not all CHIP clones confer equal risk. The “indeterminate potential” of CHIP also raises significant psychosocial concerns for patients, including increased anxiety, uncertainty, and psychological distress. Therefore, it is imperative to establish a robust framework for genetic counseling to ensure informed consent and to ethically address the management of psychosocial burdens associated with CHIP detection.

In conclusion, this study makes an important contribution to our understanding of the association between CHIP and breast cancer risk. Further investigation into the mechanisms through which CHIP mutations disrupt immune function and reshape the TME, the cumulative effects of multiple CHIP mutations, the potential cross-disease implications of these mutations, and their clinical applicability could significantly enhance the study’s impact.

## Data Availability

All data are included in the article.
